# Research versus Teaching

**Published:** 1907-01-19

**Authors:** 


### RESEARCH VERSUS TEACHING.

If we are to accept their own definitions, the immediate responsibilities and duties of those who hold the chief places in the medical schools of our Universities and hospitals, are two in number. Of these, the one is teaching; the other, research. So far as we know, no one is the least inclined to quarrel with this statement of the case. To endeavour to add to the sum of existing knowledge, and to communicate that knowledge to the next generation, with all that pertains to these exhilarating ambitions, may therefore be accepted as the recognised and acknowledged academic programme. In these circumstances, the only criticism which can possibly arise must refer to the efficiency, or otherwise, with which that programme is carried out. On such a point the outside critic is necessarily at a disadvantage, and may more or less readily be pushed to one side. But the attention which is denied to an outsider will be readily granted to those who speak from within the charmed circle of academic privilege, and from such sources it is possible to glean some hints of the attitude assumed by some of our collegiate authorities towards their acknowledged duties.

From many utterances of the order to which reference is now made, it is obvious, even to the least interested, that in recent years there has been a marked and increasing tendency to exalt the duty of research at the expense of the teaching function. The latter, we are told, may be met by text-books, or may be left in the hands of those of junior position and experience. The special duty of the head of a department is to engage in new inquiries intended to enlarge the boundaries of known truth, and to acquire a reputation for original observation and work. From the same source, and from this alone, are to be obtained glory and praise for our academic institutions, the strength and value of which are to be constructed, not in their class-rooms, but in the private laboratories of their professors. The duty of teaching, in short, is described not merely as of entirely secondary importance, but as an irritating interference with the more urgent and noble claim of original research.

In considering the question so presented, it is not in the least necessary to say anything in depreciation of research. The importance of this is universally allowed. It is equally allowed that every man's responsibility in this direction is measured by the amount of his opportunity. Therefore, the protest of those with whose attitude we are now concerned, that much is to be expected from them, is here fully and freely admitted. But it may be questioned whether their zeal to discharge one set of duties can be held to be a valid excuse for an attempt to avoid responsibilities having at least an equal claim. It must be remembered that to a very large extent the income of our medical schools is derived from the fees of the students, and that these fees are paid by those who present themselves as pupils to be instructed in the proper business of the profession they have selected. Upon the most elementary ethical principle this money must be used for the purpose for which it is paid, and hence the very first duty of those who accept it, is to provide the teaching for which they receive the fees. If original work and research should be encouraged, and no one questions this, let them be encouraged in a declared and straightforward fashion. But no excuse can be found for a proposal to take money for one purpose and to use it for another.

Whilst, however, we say that the primary duty of those who accept students' fees is to supply the teaching for which they are paid, we, at the same time, dispute the suggestion that such a procedure is inconsistent with devotion to original work. The fact is that if a man has the root of the matter in him he will be an original observer in any circumstances, and that, speaking generally, the teachers in our medical schools have quite exceptional opportunities in this direction. They have material, they have resources. In some cases, not, alas! in all, they have a fair income, and a certain provision for the future. The amount of teaching work that is demanded from them is, at the outside, not more than an hour or two a day, and this only for about eight months of the year. To hear, in such circumstances as these, a professor publicly bewailing his inability to carry on successfully the practice of original work because of the demands made on him as a teacher, is apt to provoke cynical and sarcastic comment. Evidently what is wanted is an increased appreciation both of the importance, and of the responsibility, of teaching. This granted, there will be no desire to escape from its obligations and to pass these on to members of the junior staff. And in any event, whilst from those who have large opportunities for observation and experiment much is properly required, this expectation is not inconsistent with the demand that they should fulfil their work as teachers. Let them learn to do the one, and, also, not to leave the other undone.

